# Comparison of Inflammatory Biomarkers in Females with and Without Patellofemoral Pain and Associations with Patella Position, Hip and Knee Kinematics, and Pain

**DOI:** 10.3390/biomedicines13030761

**Published:** 2025-03-20

**Authors:** Lori A. Bolgla, Sharad Purohit, Daniel C. Hannah, David Monte Hunter

**Affiliations:** 1 Department of Physical Therapy, College of Allied Health Sciences, Augusta University, Augusta, GA 30912, USA; 2 Center for Biotechnology and Genomic Medicine, Augusta University, Augusta, GA 30912, USA; spurohit@augusta.edu; 3 Department of Orthopaedic Surgery, Medical College of Georgia, Augusta University, Augusta, GA 30912, USA; dahannah@augusta.edu (D.C.H.); mohunter@augusta.edu (D.M.H.)

**Keywords:** matrix metalloproteinase-9, anterior knee pain, osteoarthritis

## Abstract

**Background/Objectives**: Patellofemoral pain (PFP) is believed to be a precursor to knee osteoarthritis (OA). The primary purpose of this study was to compare matrix metalloproteinase-9 (MMP-9) levels in young adult females with and without PFP. The secondary purpose was to determine the associations between MMP-9, patella position, hip and knee kinematics, and pain in females with PFP. **Methods**: Plasma was analyzed for MMP-9. Patellar position was measured using diagnostic ultrasound as the degree of offset (RAB angle) from the deepest aspect of the femoral trochlear groove to the inferior pole of the patella. A positive RAB angle suggested patella lateralization. Hip and knee kinematics during a single-leg squat were measured using 2-dimensional motion analysis and quantified as the dynamic valgus index (DVI), a combined measure of hip and knee motion. A higher DVI suggests increased valgus loading at the patellofemoral joint. Pain was measured using a 10 cm visual analog scale. **Results**: Females with PFP had significantly higher levels of MMP-9 than controls (72.7 vs. 58.0 ng/mL, *p* = 0.03). Females with PFP had a significant positive association between MMP-9 and patella lateralization (*r* = 0.38, *p* = 0.04), suggesting that greater patellar lateralization may contribute to increased joint inflammation. A significant inverse association was observed between MMP-9 and the DVI (*r* = −0.50, *p* = 0.007), indicating that individuals with higher inflammatory marker levels may adopt movement patterns that reduce valgus loading. **Conclusions**: The significant association between MMP-9 and patella lateralization suggested a potential link between patella alignment and joint inflammation, which may contribute to early joint degeneration. The inverse association between MMP-9 levels and the DVI suggested that subjects with higher MMP-9 levels adjusted their movement pattern as a compensatory mechanism to reduce knee valgus stress to reduce joint degeneration.

## 1. Introduction

The 2023 Global Burden of Disease Study estimated that 595 million people globally experience osteoarthritis (OA) and projected that close to 1 billion people will have some form of OA by 2050 [1]. This group also reported that over half of the cases may occur at the knee. The results of this study align with others that reported a higher incidence of knee OA in females than males [2,3]. Unfortunately, researchers have found that knee OA can adversely affect function and quality of life [4,5,6].

Patellofemoral pain (PFP) is a common, chronic knee condition [7]. While the exact number of those with PFP is unknown [8], PFP has been reported in at least 25% of knee pain complaints [7], with some estimates as high as 45% [9]. A concern is that PFP may contribute to knee OA [10,11,12,13]. Like knee OA, females experience PFP in higher numbers than males [7]. Ongoing pain leads to reduced physical activity levels, anxiety, kinesiophobia, and catastrophizing, all of which negatively affect quality of life in females with PFP [5,14,15,16,17,18,19]. This pattern underscores the need to understand the pathophysiology of PFP.

PFP is a multifactorial problem thought to result from a loss of tissue homeostasis from excessive patellofemoral joint (PFJ) loading [20]. Excessive loading can result from various interactions between the patella and femur. Increased patella lateralization (based on a static, non-weight bearing measure) may increase lateral PFJ loading by directing ground reaction forces to the lateral patellar facet [21,22]. During weight bearing activities, altered lower extremity kinematics, like increased hip adduction, hip internal rotation, and knee valgus, can also cause increased lateral (valgus) PFJ loading [23,24]. Such kinematics have been shown to impart increased stress on the lateral PFJ [25,26].

PFP is diagnosed based on common impairments such as pain during activities that require loading on a flexed knee (e.g., running, squatting, kneeling, and stair ambulation) [7]. Many patients undergo imaging that provides limited, if any, information. Prior works have reported that degenerative changes are likely not evident on radiographs for 20 or more years after onset [27,28]. Furthermore, van der Heijden et al. [29,30] reported no association between PFP and magnetic resonance imaging (MRI) features and no differences in cartilage loss in young adults with and without PFP. These findings highlight the need for other ways to detect degenerative changes before they become evident via imaging.

Recent advances in biomarker research have identified matrix metalloproteinase-9 (MMP-9) as a potential indicator of cartilage degradation and inflammation in individuals with OA [31]. MMP-9, an enzyme upregulated by inflammatory cytokines, is elevated in individuals with OA [31,32]. Favero et al. [33] highlighted that joint synovial inflammation can predict OA prior to evident radiographic changes. More recently, Li et al. [34] examined the influence of interleukin-1 beta (IL-1β), a proinflammatory cytokine believed to cause cartilage pathology [35]. They reported that increases in IL-1β resulted in increased MMP-9 gene expression. Elevated MMP-9 can lead to OA by breaking down the extracellular matrix that includes collagen.

Evidence has suggested that PFP may be a precursor to knee OA onset [11,36], which may not be diagnosed for many years [28]. To date, researchers have not examined the presence of inflammatory biomarkers in individuals with PFP. Since PFP is a chronic condition that may lead to knee OA, it is plausible that MMP-9 may be elevated in this patient population. Understanding the role of MMP-9 may lead to improved therapeutic strategies for treating PFP as a potential way to slow and/or prevent the degenerative changes associated with OA.

The primary purpose of this study was to compare MMP-9 in females with and without PFP. The secondary purpose was to determine whether patella position, lower extremity kinematics during a single-leg squat (SLS), and/or pain were associated with MMP-9 in a subset of females with PFP. We hypothesized that females with PFP would have significantly greater MMP-9 levels than controls. We also hypothesized that a positive association would exist between MMP-9 levels and (a) static patella position, (b) hip and knee kinematics during an SLS, and (c) pain in females with PFP.

## 2. Materials and Methods

### 2.1. Research Design

An observational, cohort design was used for this investigation. To test the hypothesis for the investigation’s primary aim, all subjects provided a blood sample. To test the hypothesis for the investigation’s secondary aim, only data from the subset of subjects with PFP and a complete data set were used.

### 2.2. Subjects

**Figure 1:**
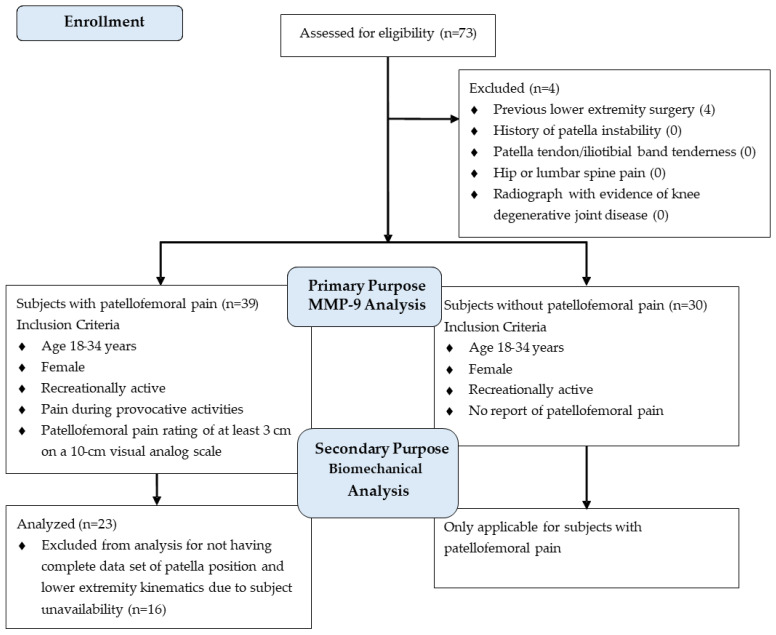
Flow of subjects through the study protocol.

An a priori power analysis was conducted using G*Power (v3.1.9.7) for a medium-to-large effect size (d = 0.65) with an α = 0.05 and β = 0.20. The power analysis suggested a minimum of 26 subjects per group were required to achieve statistical power. Subjects were recruited in the greater Central Savannah River Area by placing flyers on two campuses of a local university, at area fitness clubs, and at an academic medical center sports medicine clinic (Figure 1). Only females participated since they are more likely to experience PFP and because of the possibility of naturally occurring sex differences in MMP-9 levels [37,38,39]. In total, 39 females with PFP and 30 controls participated in the study. The subjects’ ages ranged from 18 to 34 years. This age range was selected because of an increased prevalence of OA onset after the age of 40 years [40]. Inclusion and exclusion criteria were based on prior works [38,41]. All subjects were recreationally active, defined as exercising for at least 30 min 3 times a week for at least the past 6 months. Subjects with PFP met additional criteria regarding their anterior knee pain: (a) rated at least 3 on a 10 cm visual analog scale (VAS) during daily living or recreational activities (e.g., running, walking, squatting, stair ambulation) over the previous week, (b) insidious onset for at least 4 weeks, (c) provoked by at least three of the following: during or after activity, prolonged sitting, stair ambulation, or squatting. None of the subjects with PFP had sought rehabilitation or undergone any prior movement retraining programs to improve SLS mechanics. Individuals with the following were excluded from study participation: (a) previous lower extremity surgery or significant injury, (b) recurrent patella dislocation or subluxation, (c) patella tendon or iliotibial band tenderness, and (d) hip or lumbar spine referred pain. The most painful knee was tested in subjects with PFP [38]; controls used the limb that was determined in a random fashion. Five subjects with PFP reported bilateral symptoms. Subjects were enrolled consecutively as they met the inclusion criteria and signed an informed consent document approved by the Augusta University Institutional Review Board.

Prior to data collection, all subjects received an X-ray (sagittal plane and sunrise views) to ensure that none had evidence of degenerative changes in the PFJ. An experienced orthopedic surgeon (D.M.H.), blind to the subject group, interpreted all images. No subject showed signs of degradation in the PFJ. Subjects with PFP also completed a 10 cm VAS to report their usual amount of pain during activity for the prior week [42]. Measures were recorded to the nearest 1/10th cm.

### 2.3. Plasma Collection and Measurement of MMP-9 Levels

Blood samples were collected in a plasma blood collection tube. Next, samples were centrifuged in a swinging bucket rotor at room temperature for 20 min at 1200 rpm. Afterward, plasma was aliquoted into cryovials and stored at −80 °C until processed.

Plasma levels of MMP-9 were measured using sandwich immuno-assay on an automated enzyme-linked immunosorbent assay (ELISA) (ELLA, Biotechne, Minneapolis, MN, USA). The plasma (35 µL) was diluted by adding assay buffer (35 µL), and 50 µL of the diluted plasma was added to the ELLA plate along with other reagents, as recommended by the manufacturer. The plate was then loaded into the automated ELISA for processing. The plasma MMP-9 levels were then downloaded, expressed as ng/mL, and used for statistical analysis.

### 2.4. Static Patella Position

**Figure 2:**
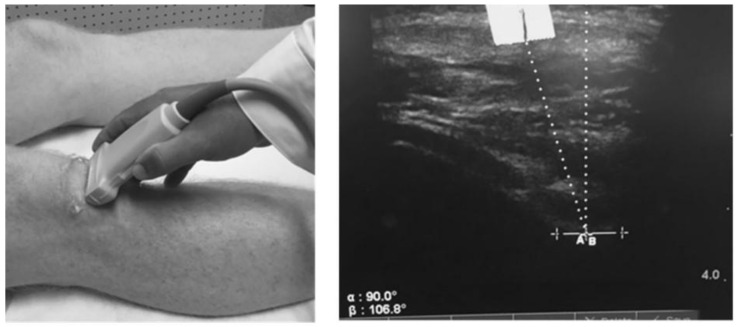
Placement of the ultrasound transducer and measurement of the patella offset (RAB) angle. The solid vertical line marker is a reference point for the interior pole of the patella. The perpendicular line represents the deepest aspect of the femoral trochlear. Courtesy of the International Journal of Sports Physical Therapy [44].

We measured the patella offset angle (RAB angle) using diagnostic ultrasound as described by Anilo et al. [43]. Briefly, Anillo et al. developed the RAB angle to quantify the patella lateralization or medialization relative to the lowest part of the femoral trochlear groove. The RAB angle was measured as follows: (1) a vertical line perpendicular to the lowest aspect of the femoral trochlea and (2) a line from the lowest aspect of the femoral trochlea to the inferior pole of the patella (Figure 2). The angle formed with the line from the lowest aspect of the femoral trochlea to the inferior patellar pole directed toward the lateral aspect of the knee represented patella lateralization. For testing, subjects were positioned supine with the quadriceps relaxed and the lower extremity in a neutral position. One examiner (D.C.H.) obtained two measurements of the test knee. All RAB angles were recorded to the nearest 1/10th of a degree; the average of the two measures was used for statistical analysis.

### 2.5. Hip and Knee Kinematics During a Single-Leg Squat

Hip and knee kinematics, collected with a 2-dimensional (2D) motion capture system (Simi Motion^®^, Unterschleinβheim, Germany), were quantified using the dynamic valgus index (DVI), a combined measure of hip and knee motion [45]. Spherical 12 mm retroreflective markers were placed on the anterior surfaces of the following landmarks: left and right anterior superior iliac spine (ASIS), the midpoint of the knee on the test extremity, and the midpoint between the medial and lateral malleolus of the ankle on the test extremity. These markers were used to measure the hip and knee frontal plane projection angles (FPPA). Markers also were placed on the greater trochanter, knee joint line, and lateral malleolus to measure knee flexion. For testing, subjects stood 2.5 m away from one camera placed in the frontal plane and another in the sagittal plane. Subjects performed the SLS barefooted. The investigator instructed the subjects to cross their arms over their chest and to squat as low as possible; they received no instruction on hip, knee, or foot position. Subjects squatted at least 50° of knee flexion (determined by visual inspection) to the beat of a metronome set at 40 beats per minute. They performed three practice and five test trials of the SLS. The motion capture system, operating at 100 Hz, recorded all data.

**Figure 3:**
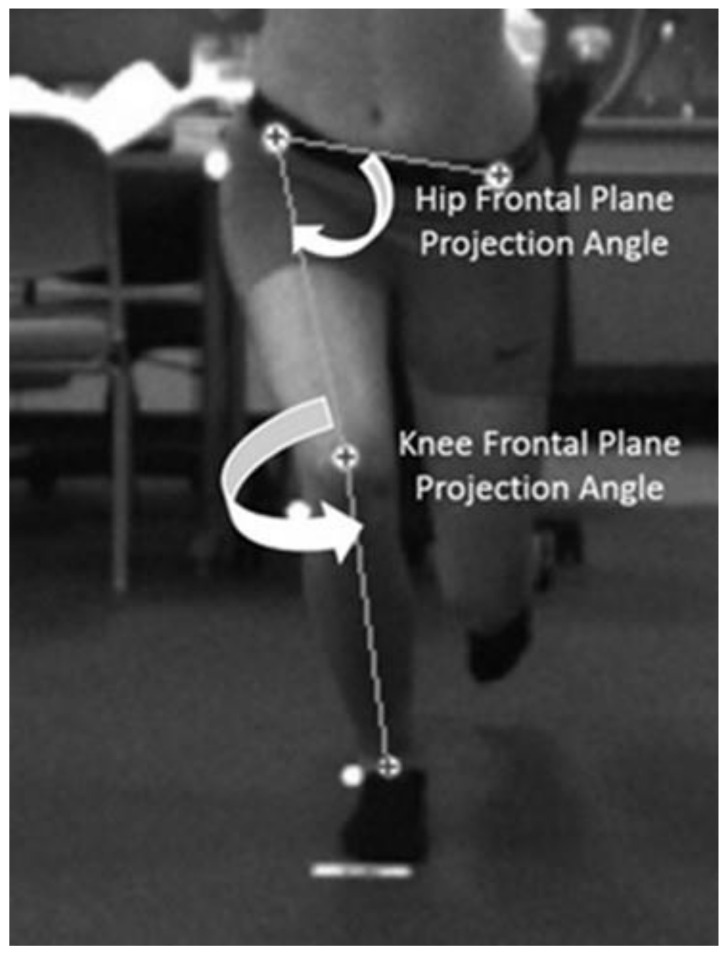
Description of the measurement of the hip frontal plane projection angle and the knee frontal plane projection angle. The dynamic valgus index is the sum of each frontal plane projection angle. Courtesy of the International Journal of Sports Physical Therapy [46].

A second-order low-pass filter, using a 6 Hz cutoff frequency, tracked and smoothed all video data. We measured the DVI at the point of peak knee flexion (the angle between the greater trochanter, lateral knee joint line, and lateral malleolus). The knee FPPA (Figure 2) was 180° minus the angle between the ASIS and the midpoint of the knee and the midpoint of the knee to the midpoint of the ankle on the test limb [45]. The DVI (Figure 3) was 90° minus the angle between the ipsilateral and contralateral ASIS and the ipsilateral ASIS and the midpoint of the distal femur (hip FPPA) plus the knee FPPA [45]. All angles were measured to the nearest 1/10th of a degree. The average of the five trials for peak DVI was used for statistical analysis.

### 2.6. Statistical Analysis

Means, standard deviations, and 95% confidence intervals (95% CI) were calculated for MMP-9 levels in all subjects. The same were calculated for RAB angles, peak DVI, and pain in the subset of subjects with PFP who completed these additional assessments. An independent *t*-test was used to compare the MMP-9 levels between females with PFP and controls. Pearson product correlation coefficients were used to determine the associations between MMP-9, RAB angle, peak DVI, and pain in the subset of subjects with PFP. We also used the Pearson product correlation to determine the associations between MMP-9, RAB angle, peak DVI, and pain in the subset of subjects with PFP and an excessive RAB angle, defined as a RAB ≥ 13 degrees [43]. All analyses were conducted using IBM SPSS Statistics for Windows, Version 28 (IBM Corp, Armonk, NY, USA); the level of significance was established at the 0.05 level. We hypothesized an increase in the measured variables; thus, we used a one-tailed test to maximize statistical power [47,48].

## 3. Results

### 3.1. Subject Characteristics

The population of interest included in this study comprised females (*n* = 69) divided into those with PFP (*n* = 39) and controls (*n* = 30) (Table 1). Females with PFP were 23.2 years old compared to controls (22.7 years). Females with PFP had a higher mass (72.8 kg vs. 64.7 kg), were shorter in height (162.3 cm vs. 165.3 cm), and reported experiencing pain (3.9 cm vs. 0 cm) (Table 1).

### 3.2. Comparison of MMP-9 Levels in Females with PFP and Control

Females with PFP (*n* = 39) had MMP-9 levels 25.6% higher than controls (*n* = 30). Average MMP-9 values for females with PFP were 72.7 (38.3) ng/mL (95% CI, 60.3–85.1) and controls were 58.0 (27.3) ng/mL (95% CI, 47.8–68.2) (Table 2).

### 3.3. Associations Between MMP-9 Levels, RAB Angle, DVI, and Pain in Females with PFP

Twenty-three subjects with PFP completed this part of the investigation. The average RAB angle was 14.6 (8.7) degrees (95% CI, 10.9–18.4), the average DVI was 34.5 (11.6) degrees (95% CI, 29.5–39.5), and the average VAS was 4.1 (1.4) cm (95% CI, 3.5–4.7) (Table 2). These females demonstrated a significant positive association between their MMP-9 levels and the RAB angle (*r* = 0.38, *p* = 0.04) and a significant inverse association between their MMP-9 levels and the DVI (*r* = −0.50, *p* = 0.007). A non-significant association (*r* = 0.12, *p* = 0.29) existed between MMP-9 levels and pain (Table 3).

For those subjects with PFP classified as having an excessive RAB angle (*n* = 14), the average RAB angle was 20.6 (6.3) degrees (95% CI, 16.7–24.4), the average DVI was 35.1 (12.1) degrees (95% CI, 27.8–42.4), and the average VAS was 4.0 (1.5) cm (95% CI, 3.1–4.9). These females demonstrated a significant positive association between their MMP-9 levels and the RAB angle (*r* = 0.52, *p* = 0.03) and a significant inverse association between their MMP-9 levels and the DVI (*r* = −0.78, *p* < 0.001). A non-significant association (*r* = 0.38, *p* = 0.09) existed between MMP-9 levels and pain (Table 4).

## 4. Discussion

PFP is a common, multifactorial problem believed to result from PFJ overload either from an altered patella position and/or faulty lower extremity kinematics [44]. More concerning is that PFP is considered a risk factor for the development of knee OA [11,12]. To date, most research directed toward understanding PFP pathology has been directed toward structural approaches (e.g., imaging and biomechanical models). This study’s uniqueness was taking a biological approach by examining MMP-9, an inflammatory biomarker, in young adult females with PFP and normal knee radiographs.

### 4.1. Comparison of MMP-9 Levels in Females with PFP and Control

Results from this study supported the primary hypothesis that females with PFP would exhibit significantly higher levels of MMP-9 than controls. This finding has provided preliminary evidence that young adult females with PFP have the elevated inflammatory biomarkers found in individuals with knee OA [31,32]. It has also afforded additional evidence that PFP is not necessarily a self-limiting condition but one of ongoing pathology [12,36,49,50]. Quantifying the degree of pathology has been a challenge for those with PFP, given the lack of useful information gained from radiographs and MRI [29,30]. None of the subjects in the current study had degenerative changes on radiographs, and this aligned with the limited usefulness of imaging. Therefore, MMP-9 may provide insight into degenerative knee changes in those with PFP prior to them becoming evident using imaging. Future studies are needed to make this determination.

To date, only two other works have examined biomarkers in those with PFP. Murphy et al. [51] compared serum cartilage oligomeric matrix protein (s-COMP), a biomarker indicative of cartilage degradation, in 18 individuals with and without chondromalacia patellae (another term used to characterize PFP). Subjects with chondromalacia patellae exhibited greater s-COMP levels compared to controls. Our previous report [44] analyzed and compared C-telopeptide fragments of type II collagen (CTX-II) in females with and without PFP and found no differences. Both investigations relied on a single biomarker to differentiate between subjects with and without PFP. Cibere et al. [52] examined biomarkers in those with knee OA and concluded that the use of this single biomarker may not have been robust enough to identify “true” degenerative changes. The paucity of available evidence supports the need for future studies aimed at identifying and understanding biomarkers associated with PFP. Most critical is the need for longitudinal studies to determine if biomarkers remain elevated over time and result in knee degenerative changes.

### 4.2. Associations Between MMP-9 Levels, Pain, RAB Angle, and DVI in Females with PFP

Results from this study partially supported these hypotheses. We hypothesized a positive association between MMP-9 and the RAB angle since increased patellar lateralization is a specific characteristic of PFJ OA [53]. Study findings (*r* = 0.38, *p* = 0.04) supported this hypothesis. We also hypothesized a positive association between MMP-9 and the DVI since an increased DVI during an SLS could cause increased valgus loads and irritation to the PFJ [45]. Although a significant association (*r* = −0.50, *p* = 0.007) existed, it was an inverse, not a positive one. A possible reason for this finding may have been that subjects with higher MMP-9 levels avoided hip adduction and knee abduction, combined motions leading to knee valgus loads, during the SLS. This finding may suggest that explaining PFP based on a strict movement-based biomechanical model may not necessarily explain PFP pathology [54]. Finally, a weak correlation (*r* = 0.12, *p* = 0.29) existed between MMP-9 and pain. No meaningful association existed between pain, the RAB, and the DVI, suggesting that these biomechanical factors were not related to pain.

A possible limitation of analyzing data for the entire cohort of females with PFP who completed the patella position and kinematics procedures may be RAB angle variability. RAB angles ranged from 1.0 to 33.5 degrees. Based on the theory that greater patella lateralization can lead to increased PFJ loading, we also examined the associations between MMP-9 levels, RAB angle, DVI, and pain in subjects (*n* = 14) with an excessive RAB angle (≥13 degrees). Results from this analysis showed a greater positive association between MMP-9 levels and the RAB and a greater inverse association between MMP-9 levels and the DVI. Though not significant, the higher positive association existed between MMP-9 levels and pain (*r* = 0.38; *p* = 0.09). Interestingly, this subgroup also had a significant inverse association between the RAB and DVI (*r* = −0.54; *p* = 0.03) and the DVI and pain (*r* = −0.48; *p* = 0.05), results that did not exist when analyzing all subjects with PFP. These correlations have provided preliminary evidence that subjects with PFP and excessive patella lateralization avoided a movement pattern (i.e., lower DVI) that could cause increased pain. This finding has highlighted the importance of assessing patella position, in combination with a dynamic movement pattern, to identify those with PFP who may have elevated MMP-9 levels. Additional studies are needed to make this determination.

It was noteworthy that the results from radiographs taken for all subjects in this investigation showed no evidence of degenerative joint disease. This finding suggested that MMP-9 levels indicative of degenerative changes were detectable earlier than imaging abnormalities. Thus, incorporating MMP-9 measurements alongside kinematic assessments (e.g., patella lateralization and increased knee valgus during weight-bearing tasks) could highlight the importance of early intervention for this cohort of females with PFP. Future work should include the analysis of other possible cartilage biomarkers, like interleukin-15 [55] and interleukin-21 [56], in combination with MMP-9.

### 4.3. Limitations

This study has limitations. First, the study only enrolled females since they are more than two times more likely to experience PFP than males [37,38]. Also, naturally occurring sex differences in MMP-9 levels could exist [39,57]. Therefore, we cannot generalize our findings to males with PFP. Another limitation is the use of a biomarker to suggest degenerative changes. While useful in monitoring disease progression, biomarkers are only an indirect method for assessing degenerative changes. However, elevated MMP-9 levels may help clinicians to identify females with PFP who may benefit from early rehabilitation [50]. Another limitation is not correlating MMP-9 levels with MRI T2 cartilage mapping. Finally, this study is cross-sectional, and it is unknown whether subjects with PFP will continue to exhibit elevated MMP-9 levels over time. Longitudinal studies are needed to determine the progression of MMP-9 levels, if any, over time and the effect on joint health.

### 4.4. Future Directions and Conclusion

A major strength of this investigation is the identification of MMP-9, an inflammatory biomarker associated with knee OA, in a cohort of young adult females with PFP. This finding highlights possible degenerative changes occurring much sooner than changes becoming evident using clinical imaging. Knowing this information supports the need for early interventions to address the impairments associated with PFP. Our results also suggest that MMP-9 could serve as a possible biomarker for diagnostic and prognostic purposes. To definitively determine whether MMP-9 is a precursor to knee OA onset, investigators should continue to examine the changes in both bone structure and MMP-9 levels over time in young adult females with PFP. Future studies should also examine males with PFP and a broader age range of individuals.

## Figures and Tables

**Table 1:** Clinical and demographic characteristics of all females with patellofemoral pain (PFP) and controls.

	PFP * *n* = 39	Controls * *n* = 30	*p*-Value ^†^
Age, y	23.2 ± 2.9	22.7 ± 3.7	0.58
Mass, kg	72.8 ± 25.8	64.7 ± 11.6	0.12
Height, cm	162.3 ± 19.8	165.3 ± 5.4	0.42
Pain, cm	3.9 ± 1.2	0.0	0.001

* Values presented are mean ± standard deviation. ^†^ Comparison made using independent *t*-test.

**Table 2:** Mean ± standard deviation for MMP-9 levels, RAB angle, dynamic valgus index (DVI), and 10 cm visual analog scale (VAS) for females with patellofemoral pain (PFP) and controls.

	PFP	Controls	*p*-Value ^†^
MMP-9, ng/mL	72.7 ± 38.3	58.0 ± 27.3	0.03
RAB angle, degrees	14.6 ± 8.7	N/A *	N/A
DVI, degrees	34.5 ± 11.6	N/A	N/A
VAS, cm	3.9 ± 1.2	0.0	0.001

^†^ Comparison made using independent *t*-test. * Not applicable since measures were only taken in the cohort of females with PFP.

**Table 3:** Correlation matrix displaying associations between MMP-9 level and RAB angle, dynamic valgus index (DVI), and visual analog scale (VAS) for females with patellofemoral pain (*n* = 23).

	MMP-9	RAB	DVI	VAS
MMP-9	1	0.38 *	−0.50 ^†^	0.12
RAB angle		1	−0.27	0.07
DVI			1	−0.13
VAS				1

* *p* = 0.04. ^†^ *p* = 0.007.

**Table 4:** Correlation matrix displaying associations between MMP-9 level and RAB angle, dynamic valgus index (DVI), and visual analog scale (VAS) for females with patellofemoral pain and a RAB angle ≥ 13 degrees (*n* = 14).

	MMP-9	RAB	DVI	VAS
MMP-9	1	0.52 *	−0.78 ^†^	0.38
RAB angle		1	−0.54 *	0.32
DVI			1	−0.48 ^β^
VAS				1

* *p* = 0.03. ^†^ *p* < 0.001. ^β^ *p* = 0.05.

## Data Availability

Data from this study are available on request to the corresponding author.

## References

[1] Steinmetz J.D., Culbreth G.T., Haile L.M., Rafferty Q., Lo J., Fukutaki K.G., Cruz J.A., Smith A.E., Vollset S.E., Brooks P.M. (2023). GBD 2021 Osteoarthritis Collaborators. Global, regional, and national burden of osteoarthritis, 1990–2020 and projections to 2050: A systematic analysis for the Global Burden of Disease Study 2021. Lancet Rheumatol..

[2] Hame S.L., Alexander R.A. (2013). Knee osteoarthritis in women. Curr. Rev. Musculoskelet. Med..

[3] Lawrence R.C., Felson D.T., Helmick C.G., Arnold L.M., Choi H., Deyo R.A., Gabriel S., Hirsch R., Hochberg M.C., Hunder G.G. (2008). Estimates of the prevalence of arthritis and other rheumatic conditions in the United States. Part II. Arthritis Rheum..

[4] Vitaloni M., Botto-van Bemden A., Sciortino Contreras R.M., Scotton D., Bibas M., Quintero M., Monfort J., Carné X., de Abajo F., Oswald E. (2019). Global management of patients with knee osteoarthritis begins with quality of life assessment: A systematic review. BMC Musculoskelet. Disord..

[5] Pazzinatto M.F., Silva D.O., Willy R.W., Azevedo F.M., Barton C.J. (2022). Fear of movement and (re)injury is associated with condition specific outcomes and health-related quality of life in women with patellofemoral pain. Physiother. Theory Pract..

[6] Glaviano N.R., Kew M., Hart J.M., Saliba S. (2015). Demographic and epidemiological trends in patellofemoral pain. Int. J. Sports Phys. Ther..

[7] Willy R.W., Hoglund L.T., Barton C.J., Bolgla L.A., Scalzitti D.A., Logerstedt D.S., Lynch A.D., Snyder-Mackler L., McDonough C.M. (2019). Patellofemoral Pain. J. Orthop. Sports Phys. Ther..

[8] Sánchez M.B., Selfe J., Callaghan M.J. (2021). The prevalence of patellofemoral pain in the Rugby League World Cup (RLWC) 2021 spectators: A protocol of a cross-sectional study. PLoS ONE.

[9] Rothermich M.A., Glaviano N.R., Li J., Hart J.M. (2015). Patellofemoral pain: Epidemiology, pathophysiology, and treatment options. Clin. Sports Med..

[10] Crossley K.M., Callaghan M.J., Linschoten R. (2016). Patellofemoral pain. Br. J. Sports Med..

[11] Eijkenboom J.F.A., Waarsing J.H., Oei E.H.G., Bierma-Zeinstra S.M.A., van Middelkoop M. (2018). Is patellofemoral pain a precursor to osteoarthritis?: Patellofemoral osteoarthritis and patellofemoral pain patients share aberrant patellar shape compared with healthy controls. Bone Joint Res..

[12] Antony B., Jones G., Jin X., Ding C. (2016). Do early life factors affect the development of knee osteoarthritis in later life: A narrative review. Arthritis Res. Ther..

[13] Brenneis M., Junker M., Sohn R., Braun S., Ehnert M., Zaucke F., Jenei-Lanzl Z., Meurer A. (2023). Patellar malalignment correlates with increased pain and increased synovial stress hormone levels-A cross-sectional study. PLoS ONE.

[14] Smith B.E., Moffatt F., Hendrick P., Bateman M., Rathleff M.S., Selfe J., Smith T.O., Logan P. (2018). The experience of living with patellofemoral pain-loss, confusion and fear-avoidance: A UK qualitative study. BMJ Open.

[15] Maclachlan L.R., Collins N.J., Hodges P.W., Vicenzino B. (2020). Psychological and pain profiles in persons with patellofemoral pain as the primary symptom. Eur. J. Pain.

[16] Hott A., Brox J.I., Pripp A.H., Juel N.G., Liavaag S. (2020). Predictors of pain, function, and change in patellofemoral pain. Am. J. Sports Med..

[17] Priore L.B., Azevedo F.M., Pazzinatto M.F., Ferreira A.S., Hart H.F., Barton C., de Oliveira Silva D. (2019). Influence of kinesiophobia and pain catastrophism on objective function in women with patellofemoral pain. Phys. Ther. Sport.

[18] Glaviano N.R., Baellow A., Saliba S. (2017). Physical activity levels in individuals with and without patellofemoral pain. Phys. Ther. Sport.

[19] Glaviano N.R., Holden S., Bazett-Jones D.M., Singe S.M., Rathleff M.S. (2022). Living well (or not) with patellofemoral pain: A qualitative study. Phys. Ther. Sport.

[20] Post W.R., Dye S.F. (2017). Patellofemoral pain: An enigma explained by homeostasis and common sense. Am. J. Orthop..

[21] Kim Y.M., Joo Y.B. (2012). Patellofemoral osteoarthritis. Knee Surg. Relat. Res..

[22] Wyndow N., Collins N., Vicenzino B., Tucker K., Crossley K. (2016). Is there a biomechanical link between patellofemoral pain and osteoarthritis? A narrative review. Sports Med..

[23] Powers C.M. (2010). The influence of abnormal hip mechanics on knee injury: A biomechanical perspective. J. Orthop. Sports Phys. Ther..

[24] Salsich G.B., Graci V., Maxam D.E. (2012). The effects of movement pattern modification on lower extremity kinematics and pain in women with patellofemoral pain. J. Orthop. Sports Phys. Ther..

[25] Powers C.M. (2003). The influence of altered lower-extremity kinematics on patellofemoral joint dysfunction: A theoretical perspective. J. Orthop. Sports Phys. Ther..

[26] Lee T.Q., Morris G., Csintalan R.P. (2003). The influence of tibial and femoral rotation on patellofemoral contact area and pressure. J. Orthop. Sports Phys. Ther..

[27] Collins N.J., Oei E.H.G., de Kanter J.L., Vicenzino B., Crossley K.M. (2019). Prevalence of radiographic and magnetic resonance imaging features of patellofemoral osteoarthritis in young and middle-aged adults with persistent patellofemoral pain. Arthritis Care Res..

[28] Poole A.R. (2002). Can serum biomarker assays measure the progression of cartilage degeneration in osteoarthritis?. Arthritis Rheum..

[29] Van der Heijden R.A., de Kanter J.L., Bierma-Zeinstra S.M., Verhaar J.A., van Veldhoven P.L., Krestin G.P., Oei E.H., van Middelkoop M. (2016). Structural abnormalities on magnetic resonance imaging in patients with patellofemoral pain: A cross-sectional case-control study. Am. J. Sports Med..

[30] Van der Heijden R.A., Oei E.H., Bron E.E., van Tiel J., van Veldhoven P.L., Klein S., Verhaar J.A., Krestin G.P., Bierma-Zeinstra S.M., van Middelkoop M. (2016). No difference on quantitative magnetic resonance imaging in patellofemoral cartilage composition between patients with patellofemoral pain and healthy controls. Am. J. Sports Med..

[31] Slovacek H., Khanna R., Poredos P., Jezovnik M., Hoppensteadt D., Fareed J., Hopkinson W. (2020). Interrelationship of Osteopontin, MMP-9 and ADAMTS4 in Patients with Osteoarthritis Undergoing Total Joint Arthroplasty. Clin. Appl. Thromb. Hemost..

[32] Zeng G.Q., Chen A.B., Li W., Song J.H., Gao C.Y. (2015). High MMP-1, MMP-2, and MMP-9 protein levels in osteoarthritis. Genet. Mol. Res..

[33] Favero M., Ramonda R., Goldring M.B., Goldring S.R., Punzi L. (2015). Early knee osteoarthritis. RMD Open.

[34] Li S., Wang H., Zhang Y., Qiao R., Xia P., Kong Z., Zhao H., Yin L. (2021). COL3A1 and MMP9 Serve as Potential Diagnostic Biomarkers of Osteoarthritis and Are Associated with Immune Cell Infiltration. Front. Genet..

[35] Yu L., Luo R., Qin G., Zhang Q., Liang W. (2023). Efficacy and safety of anti-interleukin-1 therapeutics in the treatment of knee osteoarthritis: A systematic review and meta-analysis of randomized controlled trials. J. Orthop. Surg. Res..

[36] Crossley K.M., van Middelkoop M., Barton C.J., Culvenor A.G. (2019). Rethinking patellofemoral pain: Prevention, management and long-term consequences. Best Pract. Res. Clin. Rheumatol..

[37] Boling M., Padua D., Marshall S., Guskiewicz K., Pyne S., Beutler A. (2010). Gender differences in the incidence and prevalence of patellofemoral pain syndrome. Scand. J. Med. Sci. Sports.

[38] Ferber R., Bolgla L., Earl-Boehm J.E., Emery C., Hamstra-Wright K. (2015). Strengthening of the hip and core versus knee muscles for the treatment of patellofemoral pain: A multicenter randomized controlled trial. J. Athl. Train..

[39] Mouritzen U., Christgau S., Lehmann H.J., Tankó L.B., Christiansen C. (2003). Cartilage turnover assessed with a newly developed assay measuring collagen type II degradation products: Influence of age, sex, menopause, hormone replacement therapy, and body mass index. Ann. Rheum. Dis..

[40] Cui A., Li H., Wang D., Zhong J., Chen Y., Lu H. (2020). Global, regional prevalence, incidence and risk factors of knee osteoarthritis in population-based studies. eClinicalMedicine.

[41] Gwynne C.R., Curran S.A. (2018). Two-dimensional frontal plane projection angle can identify subgroups of patellofemoral pain patients who demonstrate dynamic knee valgus. Clin. Biomech..

[42] Crossley K.M., Bennell K.L., Cowan S.M., Green S. (2004). Analysis of outcome measures for persons with patellofemoral pain: Which are reliable and valid?. Arch. Phys. Med. Rehabil..

[43] Anillo R., Villanueva E., León D., Pena A. (2009). Ultrasound diagnosis for preventing knee injuries in Cuban high-performance athletes. MEDICC Rev..

[44] Bolgla L.A., Gordon R., Sloan G., Pretlow L.G., Lyon M., Fulzele S. (2019). Comparison of patella alignment and cartilage biomarkers in young adult females with and without patellofemoral pain: A pilot study. Int. J. Sports Phys. Ther..

[45] Scholtes S.A., Salsich G.B. (2017). A dynamic valgus index that combines hip and knee angles: Assessment of utility in females with patellofemoral pain. Int. J. Sports Phys. Ther..

[46] Bolgla L.A., Gibson H.N., Hannah D.C., Curry-McCoy T. (2023). Comparison of the frontal plane projection angle and the dynamic valgus index to identify movement dysfunction in females with patellofemoral pain. Int J Sports Phys Ther.

[47] Fleming K.K., Bovaird J.A., Mosier M.C., Emerson M.R., LeVine S.M., Marquis J.G. (2005). Statistical analysis of data from studies on experimental autoimmune encephalomyelitis. J. Neuroimmunol..

[48] Hales A.H. (2024). One-tailed tests: Let’s do this (responsibly). Psychol. Methods.

[49] Roemer F.W., Kwoh C.K., Fujii T., Hannon M.J., Boudreau R.M., Hunter D.J., Eckstein F., John M.R., Guermazi A. (2018). From early radiographic knee osteoarthritis to joint arthroplasty: Determinants of structural progression and symptoms. Arthritis Care Res..

[50] Stefanik J.J., Guermazi A., Roemer F.W., Peat G., Niu J., Segal N.A., Lewis C.E., Nevitt M., Felson D.T. (2016). Changes in patellofemoral and tibiofemoral joint cartilage damage and bone marrow lesions over 7 years: The Multicenter Osteoarthritis Study. Osteoarthr. Cartil..

[51] Murphy E., FitzGerald O., Saxne T., Bresnihan B. (2002). Increased serum cartilage oligomeric matrix protein levels and decreased patellar bone mineral density in patients with chondromalacia patellae. Ann. Rheum. Dis..

[52] Cibere J., Zhang H., Garnero P., Poole A.R., Lobanok T., Saxne T., Kraus V.B., Way A., Thorne A., Wong H. (2009). Association of biomarkers with pre-radiographically defined and radiographically defined knee osteoarthritis in a population-based study. Arthritis Rheum..

[53] Kalichman L., Zhang Y., Niu J., Goggins J., Gale D., Felson D.T., Hunter D.J. (2007). The association between patellar alignment and patellofemoral joint osteoarthritis features—An MRI study. Rheumatology.

[54] Van Cant J. (2025). Unmasking the Culprit: Reframing Pain in Research and Management of Patellofemoral Pain. J. Orthop. Sports Phys. Ther..

[55] Ibrahim I., Saba E., Saad N., Mohammed D. (2019). Relation of interleukin-15 with the severity of primary knee osteoarthritis. Egypt Rheumatol..

[56] Ismail S., Ibrahim I., Saad N., Saba E. (2020). Relation of interleukin-21 with primary knee osteoarthritis severity and functional disability. World Med. J..

[57] Kolhe R., Owens V., Sharma A., Lee T.J., Zhi W., Ghilzai U., Mondal A.K., Liu Y., Isales C.M., Hamrick M.W. (2020). Sex-specific differences in extracellular vesicle protein cargo in synovial fluid of patients with osteoarthritis. Life.

